# T-Fuzzy Structure on JU-Algebra

**DOI:** 10.12688/f1000research.165402.1

**Published:** 2025-09-23

**Authors:** Selamawit Hunie Gelaw, Berhanu Assaye Alaba, Mihret Alamneh Taye

**Affiliations:** 1 Bahir Dar University Department of Mathematics, Bahir Dar, Amhara, Ethiopia; 2 Department of Mathematics, Injibara University, Injibara, Amhara, Ethiopia

**Keywords:** JU-algebra, T-norm, T-fuzzy JU-subalgebra, T-Fuzzy JU-Ideals, Cartesian product

## Abstract

**Introduction:**

This study explored the application of T-norms in fuzzy algebra, specifically by examining JU-subalgebras and JU-ideals derived from crisp JU-algebras. We investigated the properties of the T-fuzzy structures within this algebraic framework. Our work focuses on characterizing idempotent T-fuzzy JU algebras and analyzing their behavior in Cartesian products.

**Method:**

We begin by defining T-fuzzy JU-subalgebras and JU-ideals using T-norm operations. Next, we examine the structural properties of these algebraic systems through a theoretical analysis. We then studied the idempotent cases to identify their distinctive features. Finally, we prove the closure properties by constructing Cartesian products of these fuzzy structures.

**Conclusion:**

Our analysis demonstrated that idempotent T-fuzzy JU algebras possess unique structural characteristics. Furthermore, we establish that the Cartesian product of the two T-fuzzy JU-subalgebras remains a T-fuzzy JU-subalgebra, and similarly for JU-ideals. These findings extend the theoretical foundations of fuzzy algebra and suggest its potential applications in related mathematical fields.

## 1. Introduction

BCK and BCI-algebras are two important classes of logical algebras introduced in Ref. 1– 3. The class of BCK-algebra is an appropriate subclass of BCI-algebras. The concept of JU algebras was first proposed in Ref. 4 in 2020. Subsequently, in 2022 ^5^ the subalgebras and ideals of the JU-algebras are investigated. The introduction of fuzzy sets ^6^ opens the door to looking at things in different dimensions, and Ref. 7 applied this concept to group theory and introduced a fuzzy subgroup, leading to the fuzzification of different algebraic structures.

The triangular norm (T-norm) first introduced in Ref. 8. He developed generalized triangular inequalities for the statistical metric space. The T-norm is a fundamental function for solving a problem that arises from the multiple-valued logic fuzzy set theory. Following Ref. 9 introducing of T-norms into the area of fuzzy logic, numerous researchers have combined the notions of fuzzy sets and T-norms into different algebraic structures such as BCI, ^10, 11^ KU, ^12, 13^ BG, ^8^ and TM. ^14^

The JU algebra is a crisp set. The combination of fuzzy set and T-norm concepts in JU-algebra offers several advantages that make them more effective in handling uncertainty, imprecision, and complex logical relationships. This motivated us to extend the notion of T-fuzzy JU-subalgebras and T-fuzzy JU-ideals of JU-algebras and investigate the results. Furthermore, this study discusses the characteristics of the idempotent T-fuzzy JU algebras. We also prove that if the T-fuzzy JU-ideal has a finite image, then every descending chain of JU-ideals converges at a finite step, and every ascending chain of JU-ideals converges at a finite step if and only if the set of values of any T-fuzzy JU ideals is a well-ordered subset of [0 *,* 1]. Moreover, the Cartesian product of any two T-fuzzy JU-subalgebras and T-fuzzy JU-ideals of a JU-algebra also preserves its fuzzy counterpart.

## 2. Preliminaries

Definition 1. ^4^ *A JU-algebra is an algebra of* ( *X*, ◦, 1) *of type* (2, 0) *with a binary operation* ◦ *and a fixed element* 1 *, if it holds,* ∀ *x, y, z* ∈ *X:*

(a) ( *x* ◦ *y*) ◦ [( *y* ◦ *z*) ◦ ( *x* ◦ *z*)] = 1 *,
*
(b) 1 ◦ *x* = *x* *,
*
(c) *x* ◦ *y* = 1 *and* *y* ◦ *x* = 1 → *x* = *y* *,* ∀ *x*, *y* ∈ *X* *.*

In what follows, let ( *X*, ◦, 1) denote a JU-algebra unless otherwise specified. For brevity, we also refer to call *X* JU-algebra.

Lemma 1. ^4, 15^ *If X is a JU-algebra, then* *x* ◦ *x* = 1 *for any* *x* ∈ *X* *.*

Definition 2. ^5^ *A nonempty subset S of a JU-algebra X is called JU-subalgebra of X, if* *x* ◦ *y* ∈ *S*, ∀ *x*, *y* ∈ *S* *.*

Definition 3. ^5^ *A nonempty subset J of a JU-algebra X is said to be a JU-ideals of X if it satisfies:*

(a) 1 ∈ *J*
(b) *x*, *x* ◦ *y* ∈ *J* → *y* ∈ *J*, ∀ *x*, *y* ∈ *X* *.*

Definition 4. ^16, 17^ *A fuzzy set* *ϖ* *in a set X is a pair* ( *X*, *M*_*ϖ*_) *, where the function* *M*_*ϖ*_: *X* → [0, 1] *is called the memebership function of* *ϖ* *. For* *γ* ∈ [0, 1] *, the set* *U*( *M*_*ϖ*_; *γ*) = { *x* ∈ *X*| *M*_*ϖ*_ ≥ *γ*} *is called an upper level subset of* *ϖ* *.*

Definition 5. ^14^ *A triangular norm(T-norm) is a function* *T*: [0, 1] × [0, 1] → [0, 1] *that satisfies the following conditions:*

(a) *T* ( *x*, 1) = *x*
(b) *T* ( *x*, *y*) = *T* ( *y*, *x*)
(c) *T* ( *x*, *T* ( *y*, *z*)) = *T* ( *T* ( *x*, *y*), *z*)
(d) *T* ( *x*, *y*) ≤ *T* ( *x*, *z*) *whenever* *y* ≤ *z* *,* ∀ *x*, *y*, *z* ∈ [0, 1]

Examples of T-norm are:

(a) Lukasiewicz T-norm *T*_*L* *u* *k*_ ( *x*, *y*) = *m* *a* *x*{ *x* + *y* − 1, 0}, ∀ *x*, *y* ∈ [0, 1]
(b) Minimum T-norm *T*_*m* *i* *n*_ ( *x*, *y*) = *m* *i* *n*( *x*, *y*), ∀ *x*, *y* ∈ [0, 1]
(c) Product T-norm *T*_*p*_ ( *x*, *y*) = *x*. *y*, ∀ *x*, *y* ∈ [0, 1]
(d) Drastic T-norm *T*_*D*_ ( *x*, *y*) = $\left\{ \begin{array}{ll} y & \text{if}\;x=1\\ x & \text{if}\;y=1,\forall x,y\in \left[0,1\right]\\ 0 & \text{otherwise} \end{array}\right.\;$

Lemma 2. ^18^ *Let* T *and* $T^{\prime }$ *be T-norms. Then* *T*′ ( *T* ( *p*, *q*), *T* ( *r*, *s*)) = *T* ( *T*′ ( *p*, *r*), *T*′ ( *q*, *s*))

Definition 6. ^19^ *In a JU-algebra* ( *X*, ◦, 1) *, an element* *x* ∈ *X* *is idempotent if* *x* ◦ *x* = *x* *.*

Definition 7. ^14^ *Let T be a T-norm, denoted by* *T*_*i* *d* *e* *m*_ *the set of all idempotent with respect to T, That is,* *T*_*i* *d* *e* *m*_ = { *T* ( *x*, *x*) = *x* *, for some* *x* ∈ [0, 1]} *. A fuzzy set* *ϖ* *in X is said to be an idempotent T-fuzzy set if* *Im*( *M*_*ϖ*_) ⊆ *T*_*i* *d* *e* *m*_.

## 3. T-fuzzy JU-subalgebra of JU-algebra

In this section, we introduce the notion of T-fuzzy JU-subalgebra and discuss some of their properties.

Definition 8. *Let* *ϖ* = ( *X*, *M*_*ϖ*_) *be a fuzzy set in X. Then the set* *ϖ* *is a T-fuzzy JU-subalgebra with the binary operation* ◦ *if*

$$M_{\varpi }\left(x◦y\right)\geq T\left\{M_{\varpi }\left(x\right),M_{\varpi }\left(y\right)\right\},\forall x,y\in X.$$

Example 1. *Let X =* {1, 2, 3, 4} *in which* ◦ *is defined by the following Cayley table: “See* ( Table 1) ^4^ *is a JU-algebra”. Let T*: [0, 1] × [0, 1] → [0, 1] *be a function. The given T-norm defined by* *T* ( *x*, *y*) = *max*{ *x* + *y* − 1, 0} *,* ∀ *x*, *y* ∈ [0, 1] *. Define a fuzzy set* *ϖ* *in X by* *M*_*ϖ*_ (1) = 0.8 *,* *M*_*ϖ*_ (2) = 0.6 *,* *M*_*ϖ*_ (3) = 0.5 *and* *M*_*ϖ*_ (4) = 0.3 *. By routine calculation* *ϖ* *is a T-fuzzy JU-subalgebra of X.*

**Table 1.** T-fuzzy JU-subalgebra of a JU-algbera *X*.

◦	**1**	**2**	**3**	**4**
**1**	1	2	3	4
**2**	2	1	2	2
**3**	1	2	1	3
**4**	1	2	1	1

Theorem 3. *If* *ϖ* *is an idempotent T-fuzzy JU-subalgebra of X, then* *M*_*ϖ*_ (1) ≥ *M*_*ϖ*_ ( *x*).

Proof.
Suppose *ϖ* is an idempotent T-fuzzy JU-subalgebra. Since *M*_*ϖ*_ (1) = *M*_*ϖ*_ ( *x* ◦ *x*) ≥ *T*{ *M*_*ϖ*_ ( *x*), *M*_*ϖ*_ ( *x*)} = *M*_*ϖ*_ ( *x*).

Theorem 4. *The intersection of any two T-fuzzy JU-subalgebras of X is also a T-fuzzy JU-subalgebra of X.*

Proof.
Suppose *ϖ*_1_ = ( *X*, *M*_*ϖ*_1__) and *ϖ*_2_ = ( *X*, *M*_*ϖ*_2__) are two T-fuzzy JU-subalgebras of *X* and ∀ *x*, *y* ∈ *X*. Then,

$$\begin{array}{l} M_{\varpi_{1}\cap \varpi_{2}}\left(x◦y\right)=\min\left\{M_{\varpi_{1}}\left(x◦y\right),M_{\varpi_{2}}\left(x◦y\right)\right\}\\ \;\geq \min\left\{T\{M_{\varpi_{1}}\left(x\right),M_{\varpi_{1}}\left(y\right)\},T\left\{M_{\varpi_{2}}\left(x\right),M_{\varpi_{2}}\left(y\right)\right\}\right\}\\ \;\geq T\left\{\min\{M_{\varpi_{1}}\left(x\right),M_{\varpi_{2}}\left(x\right)\},\min\left\{M_{\varpi_{1}}\left(y\right),M_{\varpi_{2}}\left(y\right)\right\}\right\}\\ \;=T\left\{M_{\varpi_{1}\cap \varpi_{2}}\left(x\right),M_{\varpi_{1}\cap \varpi_{2}}\left(y\right)\right\} \end{array}$$

Corollary 5. *Let* { _*j*_: *j* ∈ Ω} *be a family of T-fuzzy JU-subalgebras of X. Then* ∩ _*j* ∈Ω_ *ϖ*_*j*_ *is also T-fuzzy JU-subalgebra of X, where* ∩ _*j* ∈Ω_ *ϖ*_*j*_ = {( *x*, *i* *n* *f*_*j* ∈Ω_ *M*_*ϖ _j_*_) | *x* ∈ *X*}.

Remark 1. *The union of any two T-fuzzy JU-subalgebras of a JU-algebra X may not be a T-fuzzy JU-subalgebra of X.*

Example 2. *Let X =* {1, 2, 3, 4} *in which* ◦ *is defined by the following Cayley table:* “( *See* Table 2) ^4^ *is a JU-algebra”. Let T*: [0, 1] × [0, 1] → [0, 1] *be a function. The given T-norm defined by* *T* ( *x*, *y*) = *min*{ *x*, *y*} *,* ∀ *x*, *y* ∈ [0, 1] *. Let us define the fuzzy set* *ϖ*_1_ *and* *ϖ*_2_ *in X by*

**Table 2.** Union of two T-fuzzy JU-subalgebra of a JU-algbra *X*.

◦	**1**	**2**	**3**	**4**
**1**	1	2	3	4
**2**	1	1	4	1
**3**	1	1	1	1
**4**	1	4	4	1

*M*_*ϖ*_1__ (1) = 0.7 *,* *M*_*ϖ*_1__ (2) = 0.4 *,* *M*_*ϖ*_1__ (3) = 0.2 *,* *M*_*ϖ*_1__ (4) = 0.1 *and*

*M*_*ϖ*_2__ (1) = 0.8 *,* *M*_*ϖ*_2__ (2) = 0.6 *,* *M*_*ϖ*_2__ (3) = 0.3 *and* *M*_*ϖ*_2__ (4) = 0.2 *.*

*Then,* *M*_*ϖ*_1___∪__*ϖ*_2__ (2 ◦ 3) = *max*{ *M*_*ϖ*_1__ (2 ◦ 3), *M*_*ϖ*_2__ (2 ◦ 3)} = 0.2                (*)

*And,* *M*_*ϖ*_1___∪__*ϖ*_2__ (2 ◦ 3) = *max*{ *M*_*ϖ*_1__ (2 ◦ 3), *M*_*ϖ*_2__ (2 ◦ 3)}

         ≥ *max*{ *T* ( *M*_*ϖ*_1__ (2), *M*_*ϖ*_1__ (3)), *T* ( *M*_*ϖ*_2__ (2), *M*_*ϖ*_2__ (3))}

         = *T*{ *max*{ *M*_*ϖ*_1__ (2), *M*_*ϖ*_2__ (2)}, *max*{ *M*_*ϖ*_1__ (3), *M*_*ϖ*_2__ (3)}} = 0.3         (**)

*From* (∗) *and* (∗∗) *we get* 0.2 ≥ 0.3 *which is false.*

Theorem 6. *Let S be a nonempty subest of a JU-algebra X. Then the characteristics function* *X*_*S*_ *is a T-fuzzy JU-subalgebras of X if and only if S is a subalgebra of X.*

Proof.
Suppose *X*_*S*_ is a T-fuzzy JU-subalgebras of *X* and *S* ≠ ∅. Let *x*, *y* ∈ *S* implies that

*X*_*S*_ ( *x*) = 1 = *X*_*S*_ ( *y*). Now

*X*_*J*_ ( *x* ◦ *y*) ≥ *T*{ *X*_*S*_ ( *x*), *X*_*S*_ ( *y*)} = *T*{1, 1} = 1

→ *X*_*S*_ ( *x* ◦ *y*) ≥ 1 but *X*_*S*_ ( *x* ◦ *y*) ≤ 1

→ *X*_*S*_ ( *x* ◦ *y*) = 1

→ *x* ◦ *y* ∈ *S*

→ *S* is the subalgebra of *X*.

Conversely, suppose *S* is a JU-subalgebra of *X*. We need to show that *X*_*S*_ is a T-fuzzy JU-subalgebra of *X*. Now consider the following cases:

Case 1: if *x*, *y* ∈ *X* where *x* ◦ *y* ∈ *S* then

*X*_*S*_ ( *x* ◦ *y*) = 1 ≥ *T*{ *X*_*S*_ ( *x*), *X*_*S*_ ( *y*)}

Case 2: if *x* ∈ *S* and *y* ∉ (or *x* ∉ *S* and *y* ∈ *S*), then

*X*_*S*_ ( *x*) = 1, *X*_*S*_ ( *y*) = 0. Thus

*X*_*S*_ ( *x* ◦ *y*) ≥ 0 = *T*{1, 0} = *T*{0, 1} = *T*{ *X*_*S*_ ( *x*), *X*_*S*_ ( *y*)}

Case 3: Suppose *x*, *y* ∉ *S* then

*X*_*S*_ ( *x*) = 0 = *X*_*S*_ ( *y*). Thus

*X*_*S*_ ( *x* ◦ *y*) ≥ 0 = *T*{0, 0} = *T*{ *X*_*S*_ ( *x*), *X*_*S*_ ( *y*)}

Theorem 7. *Let* *U*( *M*_*ϖ*_: *γ*) *be nonempty and* ∀ *γ* ∈ [0, 1] *. A fuzzy subset* *ϖ* *of a JU-algebra X is T-fuzzy JU-subalgebra of X, if and only if* *U*( *M*_*ϖ*_: *γ*) *is subalgebra of X.*

Proof.
Suppose that *ϖ* is T-fuzzy JU-subalgebra of *X*. Since *X* is JU-algebra, then 1 ∈ *X* implies that *M*_*ϖ*_ (1) ≥ *γ*.

→1∈U(M:γ)

$\to U\left(M_{\varpi }:\gamma \right)\neq \emptyset ,\gamma \in \left[0,1\right].$

Let *x*, *y* ∈ *U*( *M*: *γ*). Then, *M*_*ϖ*_ ( *x*) ≥ *γ* and *M*_*ϖ*_ ( *y*) ≥ *γ*. We have

$$M_{\varpi }\;\left(x◦y\right)\geq T\left\{M_{\varpi }\;\left(x\right),M_{\varpi }\;\left(y\right)\right\}\geq \gamma$$

This implies that *x* ◦ *y* ∈ *U*( *M*_*ϖ*_: *γ*) and hence *U*( *M*_*ϖ*_: *γ*) is a subalgebra of *X*. Conversely, suppose that *U*( *M*_*ϖ*_: *γ*) is a JU-subalgebra of *X* for any *γ* ∈ [0, 1] and *U*( *M*_*ϖ*_: *γ*) ≠ ∅. Assume that *ϖ* is not T-fuzzy JU-subalgebra of *X*. Then there exist some *z*_0_, *z*_1_ ∈ *X* such that

$$M_{\varpi }\;\left(z_{0}◦z_{1}\right)<\left\{M_{\varpi }\;\left(z_{0}\right),M_{\varpi }\;\left(z_{1}\right)\right\}$$

Take *β* = $\frac{1}{2}$ [ *M*_*ϖ*_ ( *z*_0_ ◦ *z*_1_) + { *M*_*ϖ*_ ( *z*_0_), *M*_*ϖ*_ ( *z*_1_)}]

$\to M_{\varpi }\;\left(z_{0}◦z_{1}\right)<\beta <\left\{M_{\varpi }\;\left(z_{0}\right),M_{\varpi }\;\left(z_{1}\right)\right\}$

→ *z*_0_ ◦ *z*_1_ ∉ ( *M*: *β*), a contradiction, since *U*( *M*_*ϖ*_: *β*) is a subalgebra of *X*. Therefore, *M*_*ϖ*_ ( *z*_0_ ◦ *z*_1_) ≥ { *M*_*ϖ*_ ( *z*_0_), *M*_*ϖ*_ ( *z*_1_)} for any *z*_0_, *z*_1_ ∈ *X*.

Theorem 8. *Let T be a T-norm and let* *ϖ* = ( *X*, *M*_*ϖ*_) *be a fuzzy set in a JU-algebra of X with* *I*( *M*_*ϖ*_) = { *γ*_1_, *γ*_2_, …, *γ*_*n*_} *where* *γ*_*i*_ < *γ*_*j*_ *whenever* *i* > *j* *. Assume that there exist an ascending chain of subalgebra* *S*_*o*_ ⊆ *S*_1_ ⊆ *S*_2_ ⊆, …, ⊆ *S*_*n*_ = *X* *of X such that* *M*_*ϖ*_ ( ${\tilde{S}}_{m}$ ) = *γ*_*m*_ *, where* ${\tilde{S}}_{m}$ = *S*_*m*_/ *S*_*m*−1_ *for* *m* = 1, 2, 3, *n* *and* ${\tilde{S}}_{o}$ = *S*_*o*_ *. Then* *ϖ* *is a T-fuzzy JU-subalgebra of X.*

Proof.
Suppose that there exist an ascending chain of subalgebra *S*_*o*_ ⊆ *S*_1_ ⊆ *S*_2_ ⊆, …, ⊆ *S*_*n*_ = *X* of *X* such that *M*_*ϖ*_ ( ${\tilde{S}}_{m}$ ) = *γ*_*m*_, where *m* = 1, 2, 3,…, *n*. Let *x*, *y* ∈ *X* and let *x*, *y* ∈ ${\tilde{S}}_{m}$ then *M*_*ϖ*_ ( *x*) = *γ*_*m*_ = *M*_*ϖ*_ ( *y*) and *x* ◦ *y* ∈ ${\tilde{S}}_{m}$ . Now

$$M_{\varpi }\;\left(x◦y\right)\geq \gamma_{m}=\min\left\{M_{\varpi }\;\left(x\right),M_{\varpi }\;\left(y\right)\right\}\geq T\left\{M_{\varpi }\;\left(x\right),M_{\varpi }\;\left(y\right)\right\}$$

Suppose that *x* ∈ ${\tilde{S}}_{p}$ and *y* ∈ ${\tilde{S}}_{q}$ for *p* ≠ *q* without loss of of generality we may assume that *p* > *q*. Then *M*_*ϖ*_ ( *x*) = *γ*_*p*_ < *γ*_*q*_ = *M*_*ϖ*_ ( *y*) and *x* ◦ *y* ∈ $\tilde{S}$ . Thus

$$M_{\varpi }\;\left(x◦y\right)\geq \gamma_{p}=\min\left\{M_{\varpi }\;\left(x\right),M_{\varpi }\;\left(y\right)\right\}\geq T\left\{M_{\varpi }\;\left(x\right),M_{\varpi }\;\left(y\right)\right\}$$

Hence *ϖ* is a T-fuzzy JU-subalgebra of *X*.

Theorem 9. *let S be a JU-subalgebra of X and* *ϖ* *be a fuzzy set in X given by*

$$M_{\varpi }\left(x\right)=\left\{ \begin{array}{ll} p, & \;\text{if}\;x\in S\\ q, & \;\text{otherwise}\;\forall p,q\in \left[0,1\right] \end{array}\right.$$

With *p* ≥ *q*. Then *ϖ* is a *T*_*L* *u* *k*_ fuzzy JU-subalgebra of *X*. Particularly if *p* = 1 and *q* = 0 then *ϖ* is an idempotent *T*_*L* *u* *k*_ fuzzy subalgebra *X*.

Additionally, *Im*( *ϖ*) = *S*

Proof.
Let *x*, *y* ∈ *X*. Let us consider the following cases:

Case 1, If *x*, *y* ∈ *S*, then

$$\begin{array}{l} T_{\mathrm{Luk}}\left(M_{\varpi }\left(x\right),M_{\varpi }\left(y\right)\right)=T_{\mathrm{Luk}}\left(p,p\right)\\ \;=\left(2p-1,0\right)\\ \;=\left\{ \begin{array}{ll} 2p-1, & \text{if}\;p\geq 1/2\\ 0, & \text{otherwise} \end{array}\right.\\ \;\leq p\\ \;=M_{\varpi }\left(x◦y\right) \end{array}$$

Case 2, If *x*, *y* ∉ *S*, then

$$\begin{array}{l} T_{\mathrm{Luk}}\left(M_{\varpi }\left(x\right),M_{\varpi }\left(y\right)\right)=T_{\mathrm{Luk}}\left(q,q\right)\\ \;=\left(2q-1,0\right)\\ \;=\left\{ \begin{array}{ll} 2q-1, & \text{if}\;q\geq 1/2\\ 0, & \text{otherwise} \end{array}\right.\\ \;\leq q\\ \;=M_{\varpi }\left(x◦y\right) \end{array}$$

Case 3, If *x* ∈ *S* and *y* ∉ *S* (or *x* ∉ *S* and *y* ∈ *S*), then *T*_*L* *u* *k*_ (( *x*), *M*_*ϖ*_ ( *y*)) = *T*_*L* *u* *k*_ ( *p*, *q*)

$$\begin{array}{l} =\max\left(p+q-1,0\right)\\ =\left\{ \begin{array}{ll} p+q-1, & \text{if}\;p+q\geq 1\\ 0, & \text{otherwise} \end{array}\right.\\ \leq q\\ =M_{\varpi }\left(x◦y\right) \end{array}$$

Hence *ϖ* is T-fuzzy JU-subalgebra of *X*.

Suppose that *p* = 1 and *q* = 0. Then *T*_*L* *u* *k*_ ( *p*, *p*) = *m* *a* *x*{ *p* + *p* − 1, 0} = 1 = *p* and *T*_*L* *u* *k*_ ( *q*, *q*) = { *q* + *q* − 1, 0} = 0 = *q*. Thus *p*, *q* ∈ ( *T*_*i* *d* *e* *m*_), and *Im*( *M* *_ϖ_*) = *S*.

## 4. T-fuzzy JU-Ideals of JU-algebra

In this section, we introduce the notion of T-fuzzy JU-ideals in JU-algebra and discuss some of their properties.

Definition 9. *Let* *ϖ* *be a fuzzy set in X. Then the set* *ϖ* *is a T-fuzzy JU-ideal with the binary operation* ◦ *if it satisfies the following axioms:*

a) *M*_*ϖ*_ (1) ≥ *M*_*ϖ*_ ( *x*)
b) *M*_*ϖ*_ ( *y*) ≥ *T*{ *M*_*ϖ*_ ( *x*), *M*_*ϖ*_ ( *x* ◦ *y*)}, ∀ *x*, *y* ∈ *X*.

Example 3. *Let X =* {1, 2, 3, 4, 5, 6} *in which* ◦ *is defined by the following Cayley table”* ( *See* Table 3) ^4^ *is a JU-algebra”. Let T*: [0, 1] × [0, 1] → [0, 1] *be a function. The given T-norm defined by* *T* ( *x*, *y*) = *x*. *y* *, for all* *x*, *y* ∈ [0, 1] *. Define a fuzzy set* *ϖ* *in X by* *M*_*ϖ*_ (1) = 0.9 *,* *M*_*ϖ*_ (2) = 0.7 = *M*_*ϖ*_ (3) *,* *M*_*ϖ*_ (4) = 0.5 *,* *M*_*ϖ*_ (5) = 0.3 = *M*_*ϖ*_ (6) *. By routine calculation* *ϖ* *is a T-fuzzy JU-ideals of X. (i.e.,* *M*_*ϖ*_ (4) = 0.5 ≥ 0.45 = *T*{ *M*_*ϖ*_ (1), *M*_*ϖ*_ (1 ◦ 4)} = *T*{0.9, 0.5} = (0.9).(0.5))

**Table 3.** T-fuzzy JU-ideals of a JU-algebra *X*.

◦	**1**	**2**	**3**	**4**	**5**	**6**
**1**	1	2	3	4	5	6
**2**	1	1	3	3	5	6
**3**	1	1	1	2	5	6
**4**	1	1	1	1	5	6
**5**	5	5	5	5	1	1
**6**	1	1	2	1	1	1

Theorem 10. *The intersection of any two T-fuzzy JU-ideals of X is also T-fuzzy JU-ideal of X.*

Proof.
Suppose *ϖ*_1_ = ( *X*, *M*_*ϖ*_1__) and *ϖ*_2_ = ( *X*, *M*_*ϖ*_2__) are two T-fuzzy JU-ideals of *X* and ∀ *x*, *y* ∈ *X*. Then,

$$\begin{array}{l} M_{\varpi_{1}\cap \varpi_{2}}\left(1\right)=\left\{M_{\varpi_{1}}\left(1\right),M_{\varpi_{2}}\left(1\right)\right\}\\ \;\geq \left\{M_{\varpi_{1}}\left(x\right),M_{\varpi_{2}}\left(x\right)\right\}\\ \;=M_{\varpi_{1}\cap \varpi_{2}}\left(x\right) \end{array}$$

And,

$$\begin{array}{l} M_{\varpi_{1}\cap \varpi_{2}}\left(y\right)=\min\left\{M_{\varpi_{1}}\left(y\right),M_{\varpi_{2}}\left(y\right)\right\}\\ \;\geq \min\left\{T\{M_{\varpi_{1}}\left(x\right),M_{\varpi_{1}}\left(x◦y\right)\},T\{M_{\varpi_{2}}\left(x\right),M_{\varpi_{2}}\left(x◦y\right)\}\right\}\\ \;\geq T\left\{\min\{M_{\varpi_{1}}\left(x\right),M_{\varpi_{2}}\left(x\right)\},\min\{M_{\varpi_{1}}\left(x◦y\right),M_{\varpi_{2}}\left(x◦y\right)\right\}\\ \;=T\left\{M_{\varpi_{1}\cap \varpi_{2}}\left(x\right),M_{\varpi_{1}\cap \varpi_{2}}\left(x◦y\right)\right\} \end{array}$$

Corollary 11. *Let* { *ϖ*_*j*_: *j* ∈ Ω} *be a family of T-fuzzy JU-ideals of X. Then* ∩ _*j* ∈Ω_ *ϖ*_*j*_ *is also T-fuzzy JU-ideal of X, where* ∩ _*j* ∈Ω_ *ϖ*_*j*_ = {( *x*, *i* *n* *f*_*j* ∈Ω_ *M*_*ϖj*_)| *x* ∈ *X*}.

Theorem 12. *Let J be a nonempty sub of a JU-algebra X. Then the characteristics function* *X*_*J*_ *is a T-fuzzy JU-ideals of X if and only if J is an ideal of X.*

Proof.
Suppose *X*_*J*_ is a T-fuzzy JU-ideals of *X* and *J* ≠ ∅. Let *x* ∈ *J* implies that *X*_*J*_ ( *x*) = 1.

Hence, *X*_*J*_ (1) = *X*_*J*_ ( *x* ◦ *x*) ≥ *T*{ *X*_*J*_ ( *x*), *X*_*J*_ ( *x*)} = *T*{1, 1} = 1

$\to X_{J}\;\left(1\right)\geq 1\;\mathrm{but}\;X_{J}\;\left(1\right)\leq 1$

$\to X_{J}\;\left(1\right)=1$

→1∈J

And,

Let *x*, *x* ◦ *y* ∈ *J* implies that *X*_*J*_ ( *x*) = 1 = *X*_*J*_ ( *x* ◦ *y*). Now

$X_{J}\;\left(y\right)\geq T\left\{X_{J}\;\left(x\right),X_{J}\;\left(x◦y\right)\right\}=T\left\{1,1\right\}=1$

$\to X_{J}\;\left(y\right)\geq 1\;\mathrm{but}\;X_{J}\;\left(y\right)\leq 1$

$\to X_{J}\;\left(y\right)=1$

→y∈J

$\to X_{J}\;\left(y\right)=1$

→ *J* is a JU-ideal of *X*.

Conversely, suppose *J* is ideal of *X*. We need to show that *X*_*J*_ is a T-fuzzy JU-ideal of *X*. Now consider the following cases:

Case 1: if *x*, *x* ◦ *y* ∈ *J* where *y* ∈ *J* then

$X_{J}\;\left(y\right)=1\geq T\left\{X_{J}\;\left(x\right),X_{J}\;\left(x◦y\right)\right\}$

Case 2: if *x* ∈ *J* and *x* ◦ *y* ∉ (or *x* ∉ *J* and *x* ◦ *y* ∈ *J*), then

*X*_*J*_ ( *x*) = 1, *X*_*J*_ ( *x* ◦ *y*) = 0. Thus

$X_{J}\;\left(y\right)\geq 0=T\left\{1,0\right\}=T\left\{0,1\right\}=T\left\{X_{J}\;\left(x\right),X_{J}\;\left(x◦y\right)\right\}$

Case 3: if *x*, *x* ◦ *y* ∉ *J* then

*X*_*J*_ ( *x*) = 0 = *X*_*J*_ ( *x* ◦ *y*). Thus

$X_{J}\;\left(y\right)\geq 0=T\left\{0,0\right\}=T\left\{X_{J}\;\left(x\right),\;X_{J}\;\left(x◦y\right)\right\}$

Theorem 13. *Let* ( *M*_*ϖ*_: *γ*) *be nonempty and* ∀ *γ* ∈ [0, 1] *. A fuzzy subset* *ϖ* *of a JU-algebra X is T-fuzzy JU-ideal of X, if and only if* *U*( *M*_*ϖ*_: *γ*) *is JU-ideal of X.*

Proof.
Suppose *ϖ* is a T-fuzzy JU-ideal of *X*. Let *γ* ∈ [0, 1] and ( *M*_*ϖ*_: *γ*) ≠ ∅.

Let *x*, *x* ◦ *y* ∈ ( *M*_*ϖ*_: *γ*) implies that *M*_*ϖ*_ ( *x*) ≥ *γ* and *M*_*ϖ*_ ( *x* ◦ *y*) ≥ *γ*. Then

$$M_{\varpi }\;\left(y\right)\geq T\left\{M_{\varpi }\;\left(x\right),\;M_{\varpi }\;\left(x◦y\right)\right\}\geq \gamma$$

This implies that *y* ∈ ( *M*_*ϖ*_: *γ*) and hence *U*( *M*_*ϖ*_: *γ*) is a JU-ideal of *X*.

Conversely, suppose that ( *M*_*ϖ*_: *γ*) is a JU-ideal of *X* for any *γ* ∈ [0, 1] and *U*( *M*_*ϖ*_: *γ*) ≠ ∅. Assume that *ϖ* is not T-fuzzy JU-ideal of *X*. Then there exist some *z*_0_ ∈ *X* such that

$$M_{\varpi }\;\left(1\right)<M_{\varpi }\;\left(z_{0}\right).\;\text{Take}\;\beta =\frac{1}{2}\left[M_{\varpi }\;\left(1\right)+M_{\varpi }\;\left(z_{0}\right)\right]$$

$$\to M_{\varpi }\;\left(1\right)<\beta <M_{\varpi }\;\left(z_{0}\right)$$

*z*_0_ ∈ *U*( *M*: *β*) and 1 ∉ *U*( *M*_*ϖ*_: *β*), which is contradict to our assumption that *U*( *M*_*ϖ*_: *β*) is a JU-ideal of *X*. Therefore, *M*_*ϖ*_ (1) ≥ *M*_*ϖ*_ ( *x*), ∀ *x* ∈ *X*. And assume that *z*_0_, *z*_1_ ∈ *X* such that

$$M_{\varpi }\;\left(z_{1}\right)<T\left\{M_{\varpi }\;\left(z_{0}\right),M_{\varpi }\;\left(z_{0}◦z_{1}\right)\right\}$$

Take *β* = $\frac{1}{2}$ [ *M*_*ϖ*_ ( *z*_1_) + { *M*_*ϖ*_ ( *z*_0_), *M*_*ϖ*_ ( *z*_0_ ◦ *z*_1_)}]

$$\to M_{\varpi }\;\left(z_{1}\right)<\beta <T\left\{M_{\varpi }\;\left(z_{0}\right),M_{\varpi }\;\left(z_{0}◦z_{1}\right)\right\}$$

$$\to \beta >M_{\varpi }\;\left(z_{1}\right)\;\text{and}\;\beta <\left\{M_{\varpi }\;\left(z_{0}\right),M_{\varpi }\;\left(z_{0}◦z_{1}\right)\right\}$$

*z*_1_ ∉ *U*( *M*: *β*), a contradiction, since *U*( *M*_*ϖ*_: *β*) is a JU-ideal of *X*.

Therefore, *M*_*ϖ*_ ( *z*_1_) ≥ { *M*_*ϖ*_ ( *z*_0_), *M*_*ϖ*_ ( *z*_0_ ◦ *z*_1_)} for any *z*_0_, *z*_1_ ∈ *X*.

Theorem 14. *Let* *ϖ* *be an idempotent T-fuzzy JU-ideals of X. Then the set* *X*_*M*_*ϖ*__ = { *x* ∈ *X*| *M*_*ϖ*_ ( *x*) = *M*_*ϖ*_ (1)} *is an ideal of X.*

Proof.
Let *ϖ* be an idempotent T-fuzzy JU-ideals of *X*. Obviously, 1 ∈ *X*_*M*_*ϖ*__. Let *x*, *x* ◦ *y* ∈ *X*_*M*_*ϖ*__ implies that *M*_*ϖ*_ ( *x*) = *M*_*ϖ*_ (1) = *M*_*ϖ*_ ( *x* ◦ *y*). Now

$$M_{\varpi }\;\left(y\right)\geq T\left\{M_{\varpi }\;\left(x\right),M_{\varpi }\;\left(x◦y\right)\right\}=T\left\{M_{\varpi }\;\left(1\right),M_{\varpi }\;\left(1\right)\right\}=M_{\varpi }\;\left(1\right)$$

→ *M*_*ϖ*_ ( *y*) ≥ *M*_*ϖ*_ (1) but *M*_*ϖ*_ ( *y*) ≤ *M*_*ϖ*_ (1) by definition 9(a)

$\to M_{\varpi }\;\left(y\right)=M_{\varpi }\;\left(1\right)$

$\to y\in {X_{M}}_{\varpi }$

Theorem 15. *If every T-fuzzy JU-ideal* *ϖ* *of X has a finite image, then every descending chain of JU-ideals of X converges at finite steps.*

Proof.
Assume that there exists a strictly descending chain *J*_1_ ⊋ *J*_2_ ⊋ *J*_3_ … of JU-ideal of *X* which does not converge at finite step. Define a fuzzy set *ϖ* in *X* by

$$M_{\varpi }\left(x\right)=\left\{ \begin{array}{ll} \frac{n-1}{n}, & \text{if}\;x\in J_{n\backslash J_{n+1}}\\ 1, & x\in \cap_{n=1}^{\infty }J_{n} \end{array},\right.$$

where *n* ∈ *N* and *J*_1_ = *X*.

Since 1 ∈ *J*_*n*_, ∀ n, *M*_*ϖ*_ (1) = 1 ≥ *M*_*ϖ*_ ( *x*), ∀ *x* ∈ *X*.

And for any *x*, *y* ∈ *X* then by the above assumption consider the following cases.

Case 1. If *x* ∈ *J*_*n*_ \ *J*_*n*+1_ and *x* ◦ *y* ∈ *J*_*m*_ \ *J*_*m*+1_ for *n* = 1, 2, 3, …; *m* = 1, 2, 3, … Without loss of generality, we may assume that *n* ≤ *m*.

Then *x* and *x* ◦ *y* ∈ *J*_*n*_, (since *J*_*m*_ ⊊ *J*_*n*_). Thus *y* ∈ *J*_*n*_ since *J*_*n*_ is a JU-ideals of *X*. Hence,

$$M_{\varpi }\;\left(y\right)\geq \frac{n-1}{n}=T\left\{M_{\varpi }\;\left(x\right),M_{\varpi }\;\left(x◦y\right)\right\}.$$

Case 2. If *x*, *x* ◦ *y* ∈ $\cap_{n=1}^{\infty }$ *J*_*n*_, then *y* ∈ $\cap_{n=1}^{\infty }$ *J*_*n*_.

Thus *M*_*ϖ*_ ( *y*) = 1 = *T*{ *M*_*ϖ*_ ( *x*), *M*_*ϖ*_ ( *x* ◦ *y*)}.

Case 3. If *x* ∉ $\cap_{n=1}^{\infty }$ *J*_*n*_ and *x* ◦ *y* ∈ $\cap_{n=1}^{\infty }$ *J*_*n*_, then there exists a positive integer *p* such that

$$x\in J_{p}\backslash J_{p+1}.$$

It follows that *y* ∈, then *M*_*ϖ*_ ( *y*) ≥ $\frac{p-1}{p}$ = *T*{ *M*_*ϖ*_ ( *x*), *M*_*ϖ*_ ( *x* ◦ *y*)}.

Or, if *x* ∈ $\cap_{n=1}^{\infty }$ *J*_*n*_ and *x* ◦ *y* ∉ $\cap_{n=1}^{\infty }$ *J*_*n*_, then there exists a positive integer *q* such that *x* ◦ *y* ∈ *J*_*q*_ \ *J*_*q*+1_. Implies that *y* ∈, then *M*_*ϖ*_ ( *y*) ≥ $\frac{q-1}{q}$ = *T*{ *M*_*ϖ*_ ( *x*), *M*_*ϖ*_ ( *x* ◦ *y*)}.

Thus, *ϖ* is a T-fuzzy JU-ideals with an infinite number of different values, which is a contradiction.

Theorem 16. *Every ascending chain of JU-ideals of X terminates at finite step if and only if the set of values of any T-fuzzy JU-ideals is a well ordered subset of* [0, 1] *.*

Proof.
Let *ϖ* be a T-fuzzy JU-ideals of *X*. Assume that the set of values of *ϖ* is not a well-ordered subset of [0, 1]. Then there exist a strictly decreasing sequence { *γ*_*n*_} such that *M*_*ϖ*_ ( *x*_*n*_) = *γ*_*n*_. This implies that ( *M*_*ϖ*_: *γ*_1_) ⊊ *U*( *M*_*ϖ*_: *γ*_2_) ⊊ *U*( *M*_*ϖ*_: *γ*_3_) ⊊ … is strictly ascending chain of the JU-ideal of *X* that does not terminate. This is impossible. In contrast, assume that a strictly ascending chain

$$J_{1}⊊J_{2}⊊J_{3}⊊\ldots .,$$ (*)

of the JU-ideals of *X* which does not converge at the finite step. Remember that *J* = ∪ _*n*∈ *N*_ *J*_*n*_ is JU-ideals of *X*. Now let us define a fuzzy set *ϖ* in *X* by

$$M_{\varpi }\left(x\right)=\left\{ \begin{array}{ll} \frac{1}{t}, & \text{if}\;t=\min\{n\in N|x\in J_{n}\\ 0, & \text{if}\;x\;\notin J_{n} \end{array}\right.$$

We went to show that *ϖ* is a T-fuzzy JU-ideal of *X*. Since 1 ∈ *J*_*n*_, ∀ *n*, *M*_*ϖ*_ (1) ≥ $\frac{1}{n}$ = *M*_*ϖ*_ ( *x*), ∀ *x* ∈ *X*.

And for any *x*, *y* ∈ *X* then by the above assumption consider the following cases.

Case 1: if *x*, *x* ◦ *y* ∈ *J*_*n*_ \ *J*_*n*−1_ for *n* = 2, 3, … then *y* ∈ *J*_*n*_ \ *J*_*n*−1_ implies that $M_{\varpi }\;\left(y\right)\geq \frac{1}{n}=T\left\{M_{\varpi }\;\left(x\right),M_{\varpi }\;\left(x◦y\right)\right\}$

Case 2: If *x* ∈ *J*_*n*_ and *x* ◦ *y* ∈ *J*_*n*_ \ *J*_*m*_ and *x* ◦ *y* ∈ *J*_*n*_ for any *m* < *n*. Since *ϖ* is a JU-ideal of *X*, then *y* ∈ *J*_*n*_.Thus *M*_*ϖ*_ ( *y*) ≥ $\frac{1}{n}$ ≥ $\frac{1}{m+1}$ ≥ *M*_*ϖ*_ ( *x* ◦ *y*).

Or, If *x* ∈ *J*_*n*_ \ *J*_*m*_ and *x* ◦ *y* ∈ *J*_*n*_ and *x* ∈ *J*_*n*_ for any *m* < *n*. Since *ϖ* is a JU-ideal of *X*, then *y* ∈ *J*_*n*_.Thus *M*_*ϖ*_ ( *y*) ≥ $\frac{1}{n}$ ≥ $\frac{1}{m+1}$ ≥ *M*_*ϖ*_ ( *x* ◦ *y*).

Therefore, *ϖ* is a T-fuzzy JU-ideals of *X*. Since the sequence (*) is not converge, *ϖ* has a strictly descending sequence of values. This contradicts that the value set of any T-fuzzy JU-ideal is well-ordered.

## 5. Cartesian product of T-fuzzy JU-algebras

In this section, the Cartesian products of the T-fuzzy JU-subalgebras and T-fuzzy JU-ideals of *X* are discussed and their properties are investigated.

Definition 10. *Let* *ϖ*_1_ = ( *X*, *M*_*ϖ*_1__) *and* *ϖ*_2_ = ( *Y*, *M*_*ϖ*_2__) *be two T-fuzzy JU-subalgebra of a JU-algebra X and Y respectively. The Cartesian product of* *ϖ*_1_ *and* *ϖ*_2_ *with respect to t-norm T denoted by* $M_{{\left[\varpi_{1}\times \varpi_{2}\right]}_{T}}$ = ( *X* × *Y*, $M_{{\left[\varpi_{1}\times \varpi_{2}\right]}_{T}}$ ) *, is defined by* $M_{{\left[\varpi_{1}\times \varpi_{2}\right]}_{T}}$ ( *x*, *y*) = *T* ( *M*_*ϖ*_1__ ( *x*), *M*_*ϖ*_2__ ( *y*)) *,* ∀( *x*, *y*) ∈ *X* × *Y* *.*

Theorem 17. *The Cartesian product of any two T-fuzzy JU-subalgebras of X is also a T-fuzzy JU-subalgebra of X.*

Proof.
Suppose *ϖ*_1_ = ( *X*, *M*_*ϖ*_1__) and *ϖ*_2_ = ( *Y*, *M*_*ϖ*_2__) be two T-fuzzy JU-subalgebras of a JU-algebra *X*. Let ( *x*_1_, *x*_2_), ( *y*_1_, *y*_2_) ∈ *X* × *Y*. Then

$$\begin{array}{l} {\left[\varpi_{1}\times \varpi_{2}\right]}_{T}\left(\left(x_{1},x_{2}\right)◦\left(y_{1},y_{2}\right)\right)=M_{{\left[\varpi_{1}\times \varpi_{2}\right]}_{T}}\left((x_{1}◦y_{1}),(x_{2}◦y_{2})\right)\\ \;=T\left(M_{\varpi_{1}}\left(x_{1}◦y_{1}\right),M_{\varpi_{2}}\left(x_{2}◦y_{2}\right)\right)\\ \;\geq T\left(T\left(M_{\varpi_{1}}\left(x_{1}\right),M_{\varpi_{1}}\left(y_{1}\right)\right),T\left(M_{\varpi_{2}}\left(x_{2}\right),M_{\varpi_{2}}\left(y_{2}\right)\right)\right)\\ \;=T\left(T\left(M_{\varpi_{1}}\left(x_{1}\right),M_{\varpi_{2}}\left(x_{2}\right)\right),T\left(M_{\varpi_{1}}\left(y_{1}\right),M_{\varpi_{2}}\left(y_{2}\right)\right)\right)\\ \;=T\left(M_{{\left[\varpi_{1}\times \varpi_{2}\right]}_{T}}\left(x_{1},x_{2}\right),M_{{\left[\varpi_{1}\times \varpi_{2}\right]}_{T}}\left(y_{1},y_{2}\right)\right) \end{array}$$

Theorem 18. *Let* $\overset{n}{\underset{j=1}{\left\{X_{j}\right\}}}$ *be the finite family of JU-algebras and* *X* = $\overset{n}{\underset{j=1}{\pi }}$ *X*_*j*_ *the Cartesian product of JU-algebras of* { *X*_*j*_} *. Let* *ϖ*_*j*_ *be a T-fuzzy JU-subalgebra of X, where* 1 ≤ *j* ≤ *n* *. Then* *ϖ* = $\overset{n}{\underset{j=1}{\pi }}$ *ϖ*_*j*_ *is defined by* *M*_*ϖ*_ ( *x*_1_, *x*_2_, …, *x*_*n*_) = $M_{{\left(\overset{n}{\underset{j=1}{\pi }}\varpi_{j}\right)}_{T}}$ ( *x*_1_, *x*_2_, …, *x*_*n*_) = *T*_*n*_ ( *M*_*ϖ*_1___( *x*_1_)_, *M*_*ϖ*_2___( *x*_2_)_, …, *M*_*ϖ* *_n_*__( *x*_*n*_)_) *is also T-fuzzy JU-subalgebra of X.*

Proof.
Let *x* = ( *x*_1_, *x*_2_, …, *x*_*n*_) and *y* = ( *y*_1_, *y*_2_, …, *y*_*n*_) be any elements of *X* = $\overset{n}{\underset{j=1}{\pi }}$ *X*_*j*_. Then

$$\begin{array}{l} M_{\varpi }\left(x◦y\right)=M_{\varpi }\left(\left(x_{1},x_{2},\ldots ,x_{n}\right)◦\left(y_{1},y_{2},\ldots ,y_{n}\right)\right)\\ \;=M_{\varpi }\left(x_{1}◦y_{1},x_{2}◦y_{2},\ldots ,x_{n}◦y_{n}\right)\\ \;=T_{n}\left(M_{\varpi_{1}}\left(x_{1}◦y_{1}\right),M_{\varpi_{2}}\left(x_{2}◦y_{2}\right),\ldots ,M_{\varpi_{n}}\left(x_{n}◦y_{n}\right)\right)\\ \;\geq T_{n}\left(T\left(M_{\varpi_{1}}\left(x_{1}\right),M_{\varpi_{1}}\left(y_{1}\right)\right),T\left(M_{\varpi_{2}}\left(x_{2}\right),M_{\varpi_{2}}\left(y_{2}\right)\right),\ldots ,T\left(M_{\varpi_{n}}\left(x_{n}\right),M_{\varpi_{n}}\left(y_{n}\right)\right)\right)\\ \;=T\left(T_{n}\left(M_{\varpi_{1}}\left(x_{1}\right),M_{\varpi_{2}}\left(x_{2}\right),\ldots ,M_{\varpi_{n}}\left(x_{n}\right)\right),T_{n}\left(M_{\varpi_{1}}\left(y_{1}\right),M_{\varpi_{2}}\left(y_{2}\right),\ldots ,M_{\varpi_{n}}\left(y_{n}\right)\right)\right)\\ \;=T\left(M_{\varpi }(x_{1},x_{2},\ldots ,x_{n}),M_{\varpi }\left(y_{1},y_{2},\ldots ,y_{n}\right)\right)\\ \;=T\left(M_{\varpi }\left(x\right),M_{\varpi }\left(y\right)\right) \end{array}$$

Definition 11. *Let* *ϖ*_1_ *and* *ϖ*_2_ *be fuzzy sets in X and T be a t-norm. Then the T-fuzzy product of* *ϖ*_1_ *and* *ϖ*_2_ *denoted by*_[ *ϖ*_1___·__*ϖ*_2_]_ *, is defined by* *M*_[ *ϖ*1 · *ϖ*2]_ ( *x*) = *T* ( *M*_*ϖ*_1__ ( *x*), *M*_*ϖ*_2__ ( *x*))∀ *x* ∈ *X* *.*

*Also*_[ *ϖ*_1___·__*ϖ*_2_]_ = $M_{\left[\varpi_{2}\cdot \varpi_{1}\right]}$

Theorem 19. *Let* *ϖ*_1_ *and* *ϖ*_2_ *be T-fuzzy JU-subalgebras of X. If* *T*′ *is a T-norm which dominates T, i.e.* *T*′ ( *T* ( *p*, *q*), *T* ( *r*, *s*)) ≥ *T* ( *T*′ ( *p*, *r*), *T*′ ( *q*, *s*)), ∀ *p*, *q*, *r*, *s* ∈ [0, 1] *. Then the* *T*′ *fuzzy product of* *ϖ*_1_ *and* *ϖ*_2_ *,* [ *ϖ*_1_ . *ϖ*2]′ *is a T-fuzzy JU-subalgebra of X.*

Proof.
Let *x*, *y* ∈ *X*, then

$$\begin{array}{l} \left[\varpi_{1}\cdot \varpi_{2}\right]\prime \left(x◦y\right)=T^{\prime }\left(M_{\varpi_{1}}\left(x◦y\right),M_{\varpi_{2}}\left(x◦y\right)\right)\\ \;\geq T^{\prime }\left(T\left(M_{\varpi_{1}}\left(x\right),M_{\varpi_{1}}\left(y\right)\right),T\left(M_{\varpi_{2}}\left(x\right),M_{\varpi_{2}}\left(y\right)\right)\right)\\ \;\geq T\left(T^{\prime }\left(M_{\varpi_{1}}\left(x\right),M_{\varpi_{2}}\left(x\right)\right),T^{\prime }\left(M_{\varpi_{1}}\left(y\right),M_{\varpi_{2}}\left(y\right)\right)\right)\;\left(\mathrm{by}\;\text{Lemma}\;2\right)\\ \;=T\left({\left[\varpi_{1}\cdot \varpi_{2}\right]}^{\prime }\left(x\right),M_{{\left[\varpi_{1}\cdot \varpi_{2}\right]}^{\prime }}\left(y\right)\right) \end{array}$$

Theorem 20. *The Cartesian product of any two T-fuzzy JU-ideals of X is also a T-fuzzy JU-ideal of X.*

Proof.
Suppose *ϖ*_1_ = ( *X*, *M*_*ϖ*_1__) and *ϖ*_2_ = ( *Y*, *M*_*ϖ*_2__) be two T-fuzzy JU-ideals of a JU-algebra *X*. Then let *x*, *y* ∈ *X* × *Y*. Now

$$\begin{array}{l} {\left[\varpi_{1}\times \varpi_{2}\right]}_{T}\left(1,1\right)=T\left(M_{\varpi_{1}}\left(1\right),M_{\varpi_{2}}\left(1\right)\right)\\ \;\geq T\left(M_{\varpi_{1}}\left(x\right),M_{\varpi_{2}}\left(y\right)\right)\\ \;={\left[\varpi_{1}\times \varpi_{2}\right]}_{T}\left(x,y\right) \end{array}$$

And, let ( *x*_1_, *x*_2_), ( *y*_1_, *y*_2_) ∈ *X* × *Y*. Then

$$\begin{array}{l} {\left[\varpi_{1}\times \varpi_{2}\right]}_{T}\left(y_{1},y_{2}\right)=T\left(M_{\varpi_{1}}\left(y_{1}\right),M_{\varpi_{2}}\left(y_{2}\right)\right)\\ \;\geq T\left(T\left(M_{\varpi_{1}}\left(x_{1}\right),M_{\varpi_{1}}\left(x_{1}◦y_{1}\right)\right),T\left(M_{\varpi_{2}}\left(x_{2}\right),M_{\varpi_{2}}\left(x_{2}◦y_{2}\right)\right)\right)\\ \;=T\left(T\left(M_{\varpi_{1}}\left(x_{1}\right),M_{\varpi_{2}}\left(x_{2}\right)\right),T\left(M_{\varpi_{1}}\left(x_{1}◦y_{1}\right),M_{\varpi_{2}}\left(x_{2}◦y_{2}\right)\right)\right)\\ \;=T\left\{{\left[\varpi_{1}\times \varpi_{2}\right]}_{T}(x_{1},x_{2}),M_{{\left[\varpi_{1}\times \varpi_{2}\right]}_{T}}\left((x_{1}◦y_{1}),(x_{2}◦y_{2})\right)\right\}\\ \;=T\left\{{\left[\varpi_{1}\times \varpi_{2}\right]}_{T}(x_{1},x_{2}),M_{{\left[\varpi_{1}\times \varpi_{2}\right]}_{T}}\left((x_{1}◦x_{2}),(y_{1}◦y_{2})\right)\right\} \end{array}$$

## 6. Conclusion

In this study, we introduced the concepts of T-fuzzy JU-subalgebras and T-fuzzy JU-ideas of JU-algebras and obtained important results. The characteristics of idempotent T-fuzzy JU-algebra were discussed. We prove that if every T-fuzzy JU-ideal has a finite image, then every descending chain of JU-ideal converges at finite steps and every ascending chain of JU-ideals converges at a finite step if and only if the set of values of any T-fuzzy JU-ideals is a well ordered subset of [0, 1]. Moreover, the Cartesian product of any two T-fuzzy JU-subalgebras and T-fuzzy JU-ideals of a JU-algebra are also T-fuzzy JU-subalgebra and T-fuzzy JU-ideal respectively. This introduction of the T-norm in fuzzy JU-algebras opens the door to more effective modeling of real-world problems involving uncertainty and potential topics to develop its study in the future, such as a derivatives, bipolar forms, and interval values.

## Declarations

### Ethics approval and consent to participate

Not applicable.

## Consent for publication

Not applicable.

## Data Availability

All data analyzed during this study are included in the manuscript.
