# Supplementary material for: T-Fuzzy Structure on JU-Algebra
Source: F1000Res. 2025 Sep 23;14:971. [Version 1] doi: 10.12688/f1000research.165402.1 (PMC12531617; doi:10.12688/f1000research.165402.1)
Supplement: Peer review history

# Reviewer report: Reviewer response for version 1

**DOI:** 10.5256/f1000research.182032.r421232

**Published:** 2025-10-17

**Authors:** Beza Lamesgin Derseh

**Affiliations:** 1 Debre Markos University, Debre Markos, Amhara, Ethiopia

After carefully reading the manuscript thoroughly, I have found that the manuscript introduces a new concept of T-fuzzy structures in JU-algebra **,** presenting key theoretical findings that enhance the understanding of these structures by extending existing algebraic frameworks through innovative approaches. The study explores the application of T-norms in fuzzy algebra, focusing on JU-subalgebras and JU-ideals derived from JU-algebras. It establishes definitions for T-fuzzy JU-subalgebras and JU-ideals based on T-norm operations, examines their structural characteristics, and identifies the distinctive features of idempotent T-fuzzy JU-algebras, including their behavior under Cartesian products. Generally, the study broadens the theoretical base of fuzzy algebra, provides novel perspectives, and underscores its potential applications in advancing mathematical research.

Is the work clearly and accurately presented and does it cite the current literature?

Yes

If applicable, is the statistical analysis and its interpretation appropriate?

Not applicable

Are all the source data underlying the results available to ensure full reproducibility?

Yes

Is the study design appropriate and is the work technically sound?

Yes

Are the conclusions drawn adequately supported by the results?

Yes

Are sufficient details of methods and analysis provided to allow replication by others?

Yes

Reviewer Expertise:

Fuzzy algebra, Intuitionistic fuzzy structures, Bipolar Fuzzy Structures.

I confirm that I have read this submission and believe that I have an appropriate level of expertise to confirm that it is of an acceptable scientific standard.

# Reviewer report: Reviewer response for version 1

**DOI:** 10.5256/f1000research.182032.r421229

**Published:** 2025-10-17

**Authors:** Jehad R. Kider

**Affiliations:** 1 University of Technology, Baghdad, Iraq

**REVIEW REPORT**

**Title:** **T-Fuzzy Structure on JU-Algebra**

 Thank you for selecting me for review of this manuscript.

 1.Generally, the paper is well written and has a good structure.

 2. The aim of this paper is to offer a study explored the application of T-norms in fuzzy algebra, specifically by examining JU-subalgebras and JU-ideals derived

 from crisp JU-algebras. We investigated the properties of the T-fuzzy structures within this algebraic framework.

 3.The theoretical framework is built systematically, starting from definitions to illustrative examples and theorems.

 4.Correct the line in case 2 of Theorem 6, Case 2: if x ∈ S and y *∉*  (or x *∉*  S and y ∈ S), it must be x ∈ S and y *∉*  S(or x *∉*  S and y ∈ S).

 5.The work appears to be original and free of plagiarism, and no ethical concerns are noted.

 Taking the above into consideration, I recommend the paper for indexing in your journal.

Is the work clearly and accurately presented and does it cite the current literature?

Yes

If applicable, is the statistical analysis and its interpretation appropriate?

Yes

Are all the source data underlying the results available to ensure full reproducibility?

Yes

Is the study design appropriate and is the work technically sound?

Yes

Are the conclusions drawn adequately supported by the results?

Yes

Are sufficient details of methods and analysis provided to allow replication by others?

Yes

Reviewer Expertise:

Fuzzy metric spaces, Fuzzy norned spaces, fuzzy inner product spaces.

I confirm that I have read this submission and believe that I have an appropriate level of expertise to confirm that it is of an acceptable scientific standard.

# Reviewer report: Reviewer response for version 1

**DOI:** 10.5256/f1000research.182032.r417907

**Published:** 2025-09-30

**Authors:** Tapan Senapati

**Affiliations:** 1 Southwest University, Chongqing, Chongqing, China

This paper explores the application of T-norms in the context of fuzzy algebra, focusing on JU-algebras. Specifically, the authors investigate T-fuzzy JU-subalgebras and T-fuzzy JU-ideals derived from crisp JU-algebras, aiming to expand the theoretical framework of fuzzy algebra. By characterizing idempotent T-fuzzy JU-algebras and analyzing their behavior in Cartesian products, the authors provide a valuable contribution to the field. The study primarily aims to enrich the understanding of the structural properties of these algebraic systems, ultimately enhancing their practical applications. The concepts introduced in this study serve as foundational for further research in areas that involve uncertainty and fuzzy logic.

 Key Points for Enhancing Paper Quality:

1. Some of the definitions (such as T-fuzzy JU-subalgebra and T-fuzzy JU-ideal) are a bit dense, which may cause confusion for readers unfamiliar with the topic. A more detailed explanation or simpler formulations could be beneficial, especially for the introductory sections.
2. While the paper includes a few examples, these could be expanded or illustrated more comprehensively. Visual aids, such as diagrams or flowcharts, would help readers better grasp complex concepts like the Cartesian product of T-fuzzy JU-subalgebras.
3. The literature review section could be strengthened by providing more specific methods used in this paper and those employed in similar studies. Add these papers to the references and cite them in the introduction:Atanassov’s intuitionistic fuzzy bi-normed KU-subalgebras of a KU-algebra, Atanassov’s intuitionistic fuzzy bi-normed KU-ideals of a KU-algebra. 
4. The conclusion can be strongly revised. This reviewer strongly suggests improving the flow of the conclusion section. Start with a brief explanation of the paper's goal (like the abstract), but make sure that the conclusion differs from the abstract. Provide the main findings/claims. Explain the numerical findings of the simulations. Clearly explain the significant findings and why your paper is important.
5. The introduction must clearly describe the motivation for this work and the background of the available techniques. In principle, the selected approach and theory must be properly defined, though some improvements are necessary. Please briefly explain how the references in the introduction relate to the approach you are suggesting.
6. The overall structure is logical but could be more organized for smoother transitions between sections. A brief summary or overview at the beginning of each section would guide the reader through the paper more effectively.
7. While the language is generally clear, some sections would benefit from minor revisions for grammatical consistency. Specifically, tightening sentence structure in complex mathematical descriptions could improve readability.

The authors provide a rigorous theoretical exploration of T-fuzzy JU-algebras and their applications. Their findings, particularly regarding the idempotent T-fuzzy JU-algebras and Cartesian products, are valuable contributions to the field of fuzzy algebra. With minor revisions to improve clarity, organization, and illustrative content, this paper could make a significant impact in the mathematical community and beyond.

Is the work clearly and accurately presented and does it cite the current literature?

Yes

If applicable, is the statistical analysis and its interpretation appropriate?

Yes

Are all the source data underlying the results available to ensure full reproducibility?

Yes

Is the study design appropriate and is the work technically sound?

Yes

Are the conclusions drawn adequately supported by the results?

Yes

Are sufficient details of methods and analysis provided to allow replication by others?

Yes

Reviewer Expertise:

Fuzzy algebra, Artificial Intelligence

I confirm that I have read this submission and believe that I have an appropriate level of expertise to confirm that it is of an acceptable scientific standard.
